# Trials and Tribulations of Protecting Children from Environmental Hazards

**DOI:** 10.1289/ehp.9001

**Published:** 2006-08-14

**Authors:** Bruce P. Lanphear, Jerome Paulson, Sandra Beirne

**Affiliations:** 1 Cincinnati Children’s Environmental Health Center, Cincinnati Children’s Hospital Medical Center, Departments of Pediatrics and of Environmental Health, University of Cincinnati, Cincinnati, Ohio; 2 Mid-Atlantic Center for Children’s Health and the Environment, George Washington University, School of Public Health and Health Services, Washington, DC; 3 University of Rochester School of Medicine and Dentistry, Rochester, New York

**Keywords:** assent, children, consent, controls, environmental exposure, ethics, health, prevention, policy, research

## Abstract

Society is increasingly aware of the profound impact that the environment has on children’s health. Not surprisingly, there is increasing public scrutiny about children’s exposures to environmental hazards, especially for disadvantaged children. These trends underscore the ethical imperative to develop a framework to protect children from environmental hazards. Such a framework must include regulations to test new chemicals and other potential hazards before they are marketed, a strategy to conduct research necessary to protect children from persistent hazards that are widely dispersed in their environment, stronger regulatory mechanisms to eliminate human exposures to recognized or suspected toxicants, and guidelines about the ethical conduct of research and the role of experimental trials that test the efficacy and safety of interventions to prevent or ameliorate children’s exposure to persistent toxicants or hazards that are widely dispersed in their environment.

Protecting children from environmental hazards is a daunting task. Environmental toxicants, such as lead, methylmercury, tobacco, and other pollutants covertly enter children’s body via placental transfer during fetal growth, inhalation or ingestion of house dust, soil, breast milk, and other dietary sources during early childhood (Landrigan et al. 1998; Perera et al. 2003). Exposures to these toxicants have been linked with the new “morbidities” of childhood—intellectual impairments, behavioral problems, asthma, and preterm birth (Lanphear et al. 2005b). These and innumerable other environmental chemicals can regularly be detected in young children and women of reproductive age [Centers for Disease Control and Prevention (CDC) 2005]. Respiratory toxicants are so commonplace that we accept as inevitable that over 4 million U.S. children will develop asthma (Akinbami And Schoendorf 2002)—many through exposure to airborne pollutants (Gauderman et al. 2004; Gent et al. 2003; McConnell et al. 2002). Despite a profound attachment to our own children and intense rhetoric about the value of children, society has been unwilling to invest the resources or develop regulations that are necessary to protect children from environmental hazards.

A new framework to protect children from environmental hazards is an ethical imperative. Given the increasing evidence linking children’s exposures to environmental hazards with adverse health consequences, a framework to protect children from environment hazards must include regulations to test new chemicals and other potential hazards before they are marketed. It must include a strategy to conduct research necessary to protect children from persistent hazards that are widely dispersed in their environment. It must provide a regulatory mechanism to implement policy that will eliminate human exposures to recognized and suspected toxicants. Finally, it must contain guidelines about the ethical conduct of research and the role of experimental trials that test the efficacy and safety of interventions to prevent or ameliorate children’s exposure to persistent toxicants.

## Protecting Children from Toxicants

Regulations to protect children from environmental chemicals are evolving. In the 1960s, following the epidemic of phocomelia from thalidomide, regulations were developed to protect pregnant women from exposure to teratogenic drugs—drugs that induce either structural or functional abnormalities (Hilts 2003). These regulations require premarket testing to ensure the safety and efficacy of pharmacologic agents.

Since then, it has become increasingly clear that pregnant women are often inadvertently exposed to numerous environmental teratogens (CDC 2005). Fetal and early childhood exposures to environmental toxicants, such as lead, methylmercury, polychlorinated biphenyls, and tobacco smoke, have been associated with an increased risk for premature birth, spontaneous abortions, delinquency and conduct disorder, intellectual deficits or attention deficit hyperactivity disorder (Baghurst et al. 1992; Boroja-Aburto et al. 1999; Dietrich et al 2001; Fried et al. 1998; Grandjean et al. 1997; Jaakkola et al. 2001; Jacobson and Jacobson 1996; Lanphear et al. 2005a; Longnecker et al. 2001; Needleman et al. 1979; Schantz et al. 2003; Wakschlag et al. 2002; Weitzman et al. 2002; Windham et al. 1999; Yolton 2005). Many scientists and pediatricians are increasingly troubled about the inadequacy of the regulatory framework to protect children from environmental toxicants.

From an ethical perspective, exposure of pregnant women to environmental teratogens is no different than exposure to teratogenic drugs. The vast majority of pesticides and other environmental chemicals in use have not been tested for reproductive toxicity or developmental neurotoxicity (Claudio et al. 1999, 2000). Indeed, 75% of “high production volume” chemicals, defined as chemicals that are produced at > 1 million pounds per year, lack even the most basic toxicity testing (Claudio et al. 1999, 2000). The ethical imperative to protect the fetus and children from teratogenic environmental chemicals is ultimately no different than the imperative to protect them from teratogenic drugs. To protect children from environmental toxicants and hazards, society must require premarket testing of environmental chemicals before they are marketed (Lanphear at al. 2005b).

Society’s failure to regulate chemicals for reproductive and neurodevelopmental toxicity raises profound ethical questions. In the absence of toxicity testing, we are inadvertently employing pregnant women and children as uninformed subjects to warn us of new environmental toxicants. Our regulatory system relies heavily on epidemiologic studies to identify environmental hazards, but epidemiologic studies are difficult to mount and often require years to complete (Taubes 1995). Moreover, human studies of environmental toxicants are typically observational in design and it is inherently difficult to infer causality from such studies. Paradoxically, because industry is not obligated to supply the data on developmental neurotoxicity, the costs of human disease, research, and prevention are socialized whereas the profits are privatized. Finally, once a chemical is at last deemed toxic, the large expense necessary to eliminate an exposure is invariably used as an argument that cleanup or abatement is unaffordable.

## The Role of Experimental Trials in Environmental Health Research

Once a persistent toxicant or hazard is widely disseminated in the environment, different types of research are necessary to ensure that we protect children. For recognized toxicants or hazards, our first impulse is to eliminate it. This is justified for many environmental hazards, such as installing window guards to prevent falls, reducing emissions of airborne pollutants, using safety caps for prescription drugs and banning nonpersistent pesticides. But the solution is not always so evident; indeed, even if the solution appeared obvious to experts, it may be shown years later that it was neither safe nor efficacious. The use of impermeable mattress covers that were standard therapy for asthma control for over two decades was subsequently shown to be ineffective (Woodcock et al. 2003). Efforts to protect children from asbestos and lead by abatement inadvertently increased exposure for many children (Lanphear 1998; Mossman et al. 1990). We can, despite our best intentions, cause irreparable harm to children.

Unlike studies of pharmaceutical agents, we continue to rely heavily on observational studies and expert opinion for controlling environmental threats to children. Experimental trials (or randomized controlled trials) have been underused in environmental health research. Compared with observational studies, experimental trials can provide more definitive evidence about the causal relationship of an environmental hazard with a specific disease or disability. They can also be used to test the safety and efficacy of environmental interventions to reduce children’s exposures to environmental hazards.

Controlled trials of environmental interventions, such as lead abatement, have raised considerable controversy (Mastroianni and Kahn 2002). Although these trials are usually intended to benefit disadvantaged children, they raise uncomfortable questions about why an affluent society allows children to live in substandard housing or hazardous environments. Moreover, many people assume that experimental trials of environmental toxicants would require intentionally exposing children to environmental hazards. But these trials can also be conducted by randomly assigning children to receive an intervention to reduce an existing exposure (Lanphear et al. 1999; Morgan et al. 2004; Roberts et al. 1996).

Guidelines for the ethical conduct of environmental research involving children are needed, especially for prevention trials involving disadvantaged children who are at increased risk for environmentally induced disease and disability. It is also important to define minimal risk for the average “healthy” child. It would be “unjust, a kind of societally induced double jeopardy” to allow children who live in violent neighborhoods or who face greater-than-average environmental health hazards to encounter greater risks from research than the average healthy child (Wendler 2005). Still, studies that primarily involve disadvantaged children can be done ethically when they examine hazards that predominantly affect disadvantaged children (National Research Council and Institute of Medicine 2005), but it is critical that any research findings can ultimately be translated to benefit disadvantaged children.

Not all environmental hazards should be—or need to be—studied using randomized controlled trials. Several criteria should be met before conducting a randomized controlled trial of an environmental hazard that involves children (Appendix 1). As reviewed by Glantz (2002), some have argued that it is unethical to enroll normal or healthy children in research that does not offer the prospect of benefit. It is, of course, not possible to guarantee that subjects will benefit by participating in a research study. But studies can be designed to enhance the likelihood that subjects will benefit. In a controlled trial of lead abatement, for example, the control group could receive an injury reduction intervention (Rhoads et al. 1999). Alternatively, the intervention could be delayed in the control group (Krieger et al. 2005).

### Definition of risk and benefit

Children should not be enrolled in research with greater than minimal risk unless it offers them a prospect of benefit [Department of Health and Human Services (DHHS) 1991]. Institutional review boards (IRBs) categorize studies by their potential risk and benefit. There are three primary categories of research involving children: *a*) research that involves no greater than minimal risk; *b*) research that involves greater than minimal risk, but the risk is justified by the anticipated benefit to the participants; and *c*) research that involves greater than minimal risk and no prospect of direct benefit to research participants, but the risk represents only a minor increase over minimal risk, the research involves experience reasonably commensurate with those inherent in the child’s situation, or the research is likely to yield generalizable, vitally important knowledge about a child’s disorder or condition. (DHHS 1991).

IRBs need a clearer interpretation of the standard of minimal risk (Wendler 2005). Minimal risk, which is based on the level of risk rather than the kinds of activities children ordinarily encounter, is difficult to apply. Federal regulations define minimal risk as “the risk of harm or discomfort ordinarily encountered in daily life or the performance of routine physical or psychological examinations or tests” (DHHS 1991). But the risks ordinarily encountered in daily life are typically greater than what IRBs allow for research subjects (Wendler 2005). Given this vague guidance, it is not surprising that IRBs are inconsistent in their categorization of tests as posing a “minimal risk.” In a national survey of IRB chairpersons, a single blood draw was categorized as minimal risk by 81% respondents, whereas allergy skin testing was categorized as minimal risk by only 23% of respondents (Shah et al. 2004).

### Privacy

Information that is collected about children for research purposes may require more than conventional privacy protections, especially for longitudinal studies. Outcomes that may not become manifest until years later require long-term data storage and analysis of data, years after a pregnancy is completed or a child is grown. Data cannot be completely deidentified because they will need to be linked with subsequently collected data. Given the lapses between data collection periods and children’s developing ability to understand their rights, the privacy rights of children who are participating in longitudinal studies are best protected by viewing parental permission and child assent as an ongoing process that is repeated at appropriate intervals (Fisher et al. 1996).

### Reporting results of environmental contamination and body burden

There is considerable controversy and uncertainty about whether to report individual results to study subjects. The National Bioethics Advisory Commission (1999) recommends disclosing individual results only when the findings are scientifically valid and confirmed; the findings have significant implications for the subject’s heath concerns; and a course of action to ameliorate or treat these concerns is readily available. Some ethicists, however, argue that respect for research participants requires investigators to provide individual results to study participants, except in unusual circumstances (Shalowitz and Miller 2005).

Most environmental health researchers and community advocates would agree that commonly used clinical tests or biomarkers that conform to the National Bioethics Advisory Commission’s criteria, such as blood lead concentration and skin allergy testing, should be reported promptly to families. There is, however, considerable controversy about reporting individual test results for bio-markers that are not typically used in the clinical setting or that have uncertain implications (Shalowitz and Miller 2005). In this case, academics tend to err on the side of withholding information to minimize unnecessary anxiety, whereas community advocates often argue that families should receive tests that may be indicative of harm. Should we report the results of a pesticide or other environmental chemicals found in a child’s blood to their parent when there is little known about its toxicity? Often the family can take action to reduce their child’s exposure even before we fully characterize the toxicity profile of an environmental chemical. At a minimum, we should adhere to what was promised in the informed consent or consider making individual test results available when requested.

## Ethical Guidelines for Environmental Research Involving Children

Despite an extensive literature on the ethics of conducting research on children and vulnerable populations, there are no guidelines that are specific for the ethical conduct of research on environmental hazards involving children. There are few aspects of consent, assent, and genetic testing that distinguish environmental health research from other research, but protecting children from environmental hazards does raise some unique ethical issues.

- As a society, we recognize that some of the chemicals we intentionally put into the bodies of humans (i.e., drugs) should be tested for safety and efficacy prior to marketing. Ultimately, there is no difference between exposure to environmental teratogens and pharmaceutical teratogens. For new chemicals, we should demand regulations, such as the REACH (Registration, Evaluation and Authorisation of Chemicals) Program proposed by the European Union, to ensure that comprehensive toxicity testing is conducted before a chemical is marketed and widely disseminated (Claudio et al. 1999, 2000; European Commission 2004; Goldman 2002; Lanphear et al. 2005b). There is, however, no justification for intentional dosing of healthy children with chemicals for the purpose of evaluating toxicity.
- For persistent environmental hazards that are widely disseminated in the environment, we need guidelines to clarify when randomized controlled trials are necessary to evaluate the safety and efficacy of preventive efforts. These guidelines should include recommendations about the role of data safety monitoring boards to identify adverse events and terminate a study.
- Guidelines are needed for reporting individual results of environmental contamination and biomarkers of exposure to families who are participating in a research study for both confirmed and suspected toxicants.
- For environmental health research involving children, IRBs need to ensure that community representatives or a community advisory board was involved in the design and implementation of the study (Mastroianni and Kahn 2002). IRBs should have members who have expertise in child health and community-based research (National Research Council and Institute of Medicine 2005).
- IRBs need clearer guidance for applying the minimal risk standard, especially for environmental hazards that primarily affect children from vulnerable communities.

## Conclusion

Society has an obligation to protect children from environmental toxicants and hazards. We are increasingly aware of the profound impact that environmental influences have on children’s health. There are tragic accounts of outbreaks of overt poisonings from industrial chemicals and increasing recognition that low-level exposure to environmental chemicals and pollutants are linked with disease and disability (Rogan 1995; Wigle and Lanphear 2005). Research to examine environmental influences on children is also expanding. As a result, there is increasing public scrutiny and suspicion about children’s exposures to environmental hazards, especially for disadvantaged communities. These trends underscore the inadequacy of current regulations to protect children from suspected and confirmed environmental toxicants or hazards and the need for guidelines on the ethical conduct of environmental health research involving children.

## Appendix 1. Criteria for Conducting a Randomized Controlled Trial of an Environmental Hazard Involving Children

1. Tests questions about a widely disseminated and persistent pollutant.
2. Tests questions that cannot be answered in adults.
3. Uncertainty about the safety or efficacy of environmental interventions.
4. The causal relationship of an exposure and a disease is uncertain.
5. Includes an adequate sample size to test a hypothesis.
6. Include mechanisms for communicating research findings to participants.
7. Involves community in the design and implementation of the study.
8. Includes mechanisms to ensure that legal guardians and participants are fully informed about the rationale for the trial.
